# Association between dietary inflammatory index and infertility of women; Results from RaNCD Cohort Study

**DOI:** 10.1186/s12937-023-00865-6

**Published:** 2023-07-22

**Authors:** Jalal Moludi, Negin Kamari, Mitra Darbandi, Shayan Mostafaei, Shima Moradi, Yahya Pasdar, Farid Najafi, Jafar Navabi, Amir Saber

**Affiliations:** 1 Department of Nutritional Sciences, Faculty of Nutritional Sciences and Food Technologies, Kermanshah University of Medical Sciences, Isar Sq., across from Farabi Hospital, P.O. Box 6719851552, Kermanshah, Iran; 2 Student Research Committee, Kermanshah University of Medical Sciences, Kermanshah, Iran; 3 Research Center for Environmental Determinants of Health (RCEDH), Health Institute, Kermanshah University of Medical Sciences, Kermanshah, Iran; 4 Department of Biostatistics, Faculty of Health, Kermanshah University of Medical Sciences, Kermanshah, Iran; 5 Clinical Research Development Center, Imam Khomeini Hospital, Kermanshah University of Medical Sciences, Kermanshah, Iran

**Keywords:** Infertility, Inflammation, Dietary inflammatory index, PERSIAN cohort

## Abstract

**Background:**

In recent decades, more and more attention has been paid to the influence of nutrition on reproductive health. Nevertheless, the imminent association between diet-related inflammation and the risk of infertility has not yet been established. The aim of the current study was to investigate the ability of the Dietary Inflammatory Index (DII) to estimate infertility incidence in women.

**Methods:**

This cross-sectional study was conducted using data from Ravansar non-communicable diseases (RaNCD) cohort study on 4437 participants. The DII was calculated based on the reported consumption of up to 31 food parameters measured via a validated and reproducible 118-item food-frequency questionnaire (FFQ). Multiple logistic regression analysis was applied to estimate the multivariable odds ratio (OR) adjusted for potential confounding variables.

**Results:**

Out of all participants, 411 women (9.26%) were infertile. The mean ± SD age and weight of infertile women were 43.67 ± 7.47 years and 72.86 ± 13.02 kg, respectively. Statistical analyses showed the odds ratio of infertility in the fourth quartile (pro-inflammatory diet) was 1.76 times higher than in the first quartile (anti-inflammatory diet) of DII (95% CI: 1.57–2.02).

**Conclusions:**

The findings of this study provide compelling evidence about the association between infertility and the quality of diet in women. Therefore, interventions and programs aimed at promoting a healthy lifestyle and using healthy diets can be considered as one of the effective approaches in the prevention and treatment of infertility in women.

## Introduction

Infertility is defined as a reproductive failure after at least one year of unprotected sex, which affects approximately 10–15% of women worldwide. Approximately 15.5% of married women aged 15 to 44 years in Iran are unable to become pregnant after one year of unprotected sex (infertility) [1, 2]. Although male infertility is involved in more than half of childlessness, women’s infertility is still considered a global social problem [3]. The results from the Tehran Lipid and Glucose Study showed that the prevalence of all causes of infertility is higher than that of global infertility among Iranian women [4].

Although infertility therapeutic techniques have been developed, the high cost of these methods can lead individuals to come to alternative and inexpensive treatments [5]. Most recently, the recognition of modifiable factors in the treatment of infertility has been of interest in many studies [6–9]. The role of diet and lifestyle in infertility is largely unknown. However, substantial data suggest that dietary factors affecting inflammation may have a significant role in the etiology of some types of infertility. In addition, biochemical markers of chronic inflammation, such as high concentrations of C reactive protein (CRP), have been prospectively linked to increased infertility [10, 11]. Studies have shown that diet modification, as an important component of lifestyle, seems to be a promising approach to reducing inflammation and consequently reducing the risk of infertility [12].

The dietary inflammatory index has been designed to recognize the inflammatory potential of individuals’ dietary patterns [13]. The pro-inflammatory or anti-inflammatory properties of some foods can have beneficial or harmful effects on inflammatory factors, including interleukin-1β, IL-4, IL-6, IL-10, tumor necrosis factor-alpha (TNF-α), and CRP [14–17]. In addition, most previous studies have evaluated the relationship between reproductive function and individual nutrients, foods, food groups, or diets. Systematic reviews in this area reported no significant differences in menstrual, reproductive or metabolic characteristics of different food components in the majority of included studies [18]. Besides, Stamets et al. showed that following two different low-calorie diets (high protein and high carbohydrate) improved fertility and there was no difference between the two diets [19]. In addition, another study looked at the effects of a low-carb diet, or a diet high in monounsaturated fat, compared to a standard diet. This study found that a low-carb diet increases insulin sensitivity but has no effect on circulating reproductive hormones [20].

**Table 1:** Prevalence and 95% confidence interval of fertility status based on demographic variables

Variable	Subgroup	Number	Infertility	P-value ^*^
**DII**	Q1 (<-2.02)	361	4.16 (2.52 to 6.78)	< 0.001
	Q2 (-2.02, -1.02)	568	7.30 (5.33 to 9.91)	
	Q3 (-1.03, 0.25)	984	7.52 (6.03 to 9.34)	
	Q4 (≥ 0.26)	2524	10.97 (9.81 to 12.26)	
**Educational level**	Illiterate	1521	6.25 (5.13 to 7.58)	< 0.001
	1–5 years	2119	10.01 (8.80 to 11.36)	
	6–9 years	451	13.75 (10.86 to 17.25)	
	10–12 years	238	10.92 (7.54 to 15.57)	
	≥ 13 years	108	14.81 (9.26 to 22.87)	
**Socioeconomic status**	Q1 (Poorest)	1042	8.64 (7.08 to 10.50)	0.890
	Q2	1128	9.22 (7.66 to 11.05)	
	Q3	1125	9.24 (7.68 to 11.08)	
	Q4	815	10.06 (8.17 to 12.33)	
	Q5 (Richest)	327	9.48 (6.74 to 13.17)	
**Alcohol drinking**	No	4333	9.25 (8.43 to 10.14)	0.277
	Yes	104	25.01 (2.38 to 81.99)	
**Use contraceptive**	No	1063	12.07 (9.78 to 14.34)	< 0.001
	Yes	3374	9.18 (8.66 to 9.68)	
**Residency**	City	2636	11.95 (10.77 to 13.25)	< 0.001
	Village	1801	5.33 (4.38 to 6.47)	
**Physical activity**	Light (MET < 3)	897	11.61 (9.67 to 13.88)	0.001
	Moderate (MET 3–6)	3079	9.13 (8.16 to 10.20)	
	Intense (MET ≥ 6)	461	5.64 (3.87 to 8.16)	
**Total**		**4437**	**9.26 (8.44 to 10.15)**	**-**

**Note:** *P-value was obtained by the Chi-square test and P<0.05 were considered as statistically significant

To date, no study has examined the association between DII and the risk of women’s infertility. Therefore, to illuminate this uncharted area, the current study was performed using data from a cohort study in a RaNCD cohort study to examine the relationship between women’s infertility and DII.

## Materials and methods

### Study design and participants

This cross-sectional study was performed by using data from the RaNCD cohort study. The RaNCD cohort study is part of the PERSIAN (Prospective Epidemiological Research Studies in Iran) Cohort that has been conducted to investigate non-communicable diseases in 10,047 Kurdish participants aged 35–65 years living in Ravansar city, Kermanshah Province, West Iran since 2014. Details of this study were defined in previous studies [21, 22]. All study subjects included women who participated in the RaNCD cohort study. Also, this study was approved by the ethics committee of Kermanshah University of medical sciences with code No.IR.KUMS.REC.1401.218 and conducted in accordance with the Declaration of Helsinki.

Infertility was determined based on a self-administered questionnaire about fertility history that was completed for all participant women. All women aged 20–50 years old who had the inclusion criteria and were willing to collaborate were included in the study. Written consent has been obtained from all participants. The collected data were investigated by the center supervisor and ultimately recorded after ensuring their accuracy on the same day. In case of any problem, follow-up was performed and the subjects were asked to present in the cohort center and complete the questionnaire again. In this study, the online version of the standard questionnaires was used to collect information, in accordance with the PERSIAN cohort protocol. However, the questionnaires used in this study were revised after performing a pre-pilot phase to improve their validity and reliability. The exclusion criteria were the absence of any malignancy, following a special diet, and having conditions such as neurological, hepatic, and endocrine diseases was our inclusion criteria. Among the all participants, 4764 were men and excluded from the study. Also, other excluded participants were as follows; 447 single and divorced women, 162 women with a history of hysterectomy, 105 pregnant women, 112 women who intake more than 4200 calories, and 20 who intake less than 800 calories. Finally, 4437 women (411 infertile and 4026 non-infertile) were included in this study.

### Basic data collection

Baseline questionnaires including demographic, socioeconomic status, physical activity, smoking status, alcohol intake, dietary habits, medical history, family medical history, and FFQ were completed in face-to-face interviews by a trained interviewer for all participants recruited to RaNCD.

**Table 2:** The Metabolic profile and dietary intakes of participants

Variable	Infertility (mean ± SD)		P ^*^
	No (N = 4026)	Yes (N = 411)	
Age (year)	48.42 ± 8.16	43.67 ± 7.47	< 0.001
Weight (kg)	70.27 ± 12.77	72.86 ± 13.02	< 0.001
BMI (kg/m2)	28.75 ± 4.80	29.72 ± 4.85	< 0.001
WC (cm)	98.85 ± 11.22	99.22 ± 11.10	0.531
TG (mg/dl)	131.38 ± 80.29	130.54 ± 67.90	0.838
TC (mg/dl)	188.97 ± 39.65	186.71 ± 34.80	0.266
HDL-C (mg/dl)	49.52 ± 11.30	48.72 ± 11.75	0.172
LDL-C (mg/dl)	103.03 ± 26.45	101.38 ± 23.60	0.226
FBS (mg/dl)	97.07 ± 29.24	95.91 ± 28.69	0.447
Total calorie (Kcal/d)	1912.6 ± 352.6	2004.5 ± 275.2	0.009
Carbohydrates (g/d)	310.2 ± 110.5	322.9 ± 95.7	0.003
Fat (g/d)	45.7 ± 10.8	49.4 ± 15.2	0.080
Protein (g/d)	69.9 ± 12.8	71.4 ± 13.2	0.080
Fiber (g/d)	15.8 ± 5.9	15.2 ± 4.3	0.256
Zinc (mg/d)	5.3 ± 0.3	5.8 ± 0.1	< 0.001
Calcium (mg/d)	615.8 ± 5.9	652.3 ± 18.9	0.06
Fe (mg/d)	12.9 ± 1.7	13.7 ± 1.2	0.002
B6 (mg/d)	2.11 ± 0.93	2.29 ± 0.94	< 0.001
B12 (µg/d)	5.49 ± 4.89	5.68 ± 4.76	0.449

**Note**: Values are presented as the mean ± SD for variables with normal distribution or median (interquartile) for variables without normal distribution. BMI: body Mass Index, WC: waist circumference, TG: triglycerides, TC: total cholesterol, HDL-C: high density lipoprotein, LDL-C: low density lipoprotein, FBS: fasting blood sugar. ^*^ P-value was obtained by the independent *t* test and P < 0.05 was considered as statistically significant

### Physical activity assessment

In RaNCD cohort study, physical activity was assessed by the standard questionnaire designed by PERSIAN mega cohort study with twenty two questions about the amount of daily activities. After collecting data, the participants’ responses were reported based on the metabolic equivalent of task per hour per day (MET/h per day). Detail of this questionnaire was described in the previous study [23]. Finally the total MET were categorized in three tertiles (Light-intensity activities < 3, Moderate-intensity activities: 3–6, Vigorous intensity activities: ≥ 6) [24, 25].

### Anthropometry data

Weight was measured by an InBody 770 device while the participant was wearing light and without shoes with a precision of 100 gr. Height measurement was performed by using the automatic stadiometer BSM 370 in a standing position without shoes. Body mass index (BMI) was determined by dividing the weight (kg) by squared height (meter).

**Table 3:** Characteristics of the participants according to the quartiles of DII

Variables		Q1 < -2.02	Q2 -2.02, -1.02	Q3 -1.03, 0.25	Q4 ≥ 0.26	P _Trend_^*^
Age (years)		42.12 ± 8.41	43.21 ± 8.30	44.09 ± 8.09	46.10 ± 7.85	< 0.001
Weight (kg)		67.66 ± 13.62	69.37 ± 9.44	74.65 ± 11.01	77.16 ± 13.78	< 0.001
BMI (kg/m^2^)		28.22 ± 5.24	28.63 ± 4.86	29.87 ± 4.55	29.92 ± 4.77	< 0.001
WC (cm)		97.10 ± 12.14	98.84 ± 10.82	99.10 ± 10.41	101.58 ± 11.40	0.005
TC (mg/dl)		182.74 ± 23.71	183.12 ± 32.13	187.35 ± 37.63	192.66 ± 28.17	< 0.001
LDL-C (mg/dl)		97.32 ± 29.59	101.79 ± 26.34	103.54 ± 26.23	105.17 ± 25.46	< 0.001
HDL-C (mg/dl)		51.44 ± 11.2	50.31 ± 10.74	46.6 ± 11.44	44.24 ± 11.42	< 0.001
TG (mg/dl)		128.25 ± 70.43	129.09 ± 68.04	130.11 ± 27.14	130.45 ± 86.52	0.166
FBS (mg/dl)		95.03 ± 25.31	95.19 ± 29.44	96.23 ± 30.02	96.42 ± 29.53	0.852
Smoking% (CI 95%)	No	91.64 (88.29 to 94.10)	94.28 (91.89 to 96)	95.22 (93.69 to 96.39)	96.03 (95.19 to 96.72)	0.001^**^
	Current	4.46 (2.75 to 7.16)	1.97 (1.06 to 3.63)	1.63 (1.01 to 2.64)	1.31 (0.93 to 1.84)	
	Former	3.90 (2.32 to 6.48)	3.75 (2.40 to 5.80)	3.15 (2.23 to 4.45)	2.66 (2.10 to 3.37)	
Total calorie intake (Kcal/d)		1877.52 ± 131.39	1934.95 ± 186.34	2079.93 ± 144.49	2135.58 ± 175.58	< 0.001
Carbohydrate (Kcal/d)		1287.16 ± 96.43	1286.82 ± 104.16	1289.33 ± 61.42	1294.74 ± 61.40	0.8974
Fat (Kcal/d)		439.64 ± 44.13	443.18 ± 22.52	448.68 ± 27.62	449.06 ± 32.77	0.0725
Protein (Kcal/d)		150.52 ± 14.06	204.88 ± 19.10	341.32 ± 13.49	391.78 ± 22.09	< 0.001
Fiber (g/d)		32.87 ± 2.61	24.86 ± 4.05	18.97 ± 3.27	10.07 ± 1.13	< 0.001

**Note**: Values are presented as the mean ± SD for variables with normal distribution or median (interquartile) for variables without normal distribution. Q: quartile, BMI: body Mass Index, WC: waist circumference, LDL-C: low density lipoprotein, HDL-C: high density lipoprotein, TC: total cholesterol, TG: triglycerides, FBS: fasting blood sugar, E: energy percent. ^*^ P-value was obtained by the One-way ANOVA test between quartiles (the fourth quartile compared to the first quartile) and P < 0.05 were considered as statistically significant. ^**^ P-value was obtained by chi-square test.

### Dietary assessment and DII calculation

At the RaNCD study site, a 118-item FFQ was completed, and the validity and reliability of this questionnaire have been assessed in previous studies [26, 27]. We obtained dietary information from this questionnaire and then calculated the DII score based on the method developed by Shivappa et al. [28]. In this method, 45 food items were introduced that had a role in decreasing or increasing inflammation. In this study, among these food items, we accessed only 31 of them, including onion, garlic, coffee, tea, energy, protein, carbohydrates, fiber, vitamins A, C, D, E, B_1,_ B_2,_ B_3,_ B6, B_12_, folate, beta-carotene, total fat, saturated fatty acids (SFAs), monounsaturated fatty acids (MUFAs), polyunsaturated fatty acids (PUFAs), omega 3 and 6, cholesterol, Mg, Fe, Se, and Zn. These food item intakes were linked to the world database mean and SD from 11 studies worldwide [28]. Finally, all food items specified for the DII score were summed, and the DII score was created. A higher DII adhered to a pro-inflammatory diet, and a low DII was associated with an anti-inflammatory diet. The DII quartile cutoff points were categorized based on cutoff points: Quartiles 1= < -2.02; Quartiles 2= -2.02, -1.02; Quartiles 3= -1.03, 0.25; and Quartiles 4 ≥ 0.26.

### Statistical methods

Data are accessible as the mean (standard deviation) and frequency (%) for continuous and categorical variables. The normality of the data was assessed and proven by the Kolmogorov–Smirnov test. Independent samples t-tests and one-way ANOVA tests were used for between-group differences of continuous variables. Additionally, the chi-square/Fisher exact test was used to assess the associations between two categorical variables, as well as the chi-square test was used for testing multiple categorical variables. Logistic regression was performed to calculate odds ratios (ORs) and 95% confidence intervals (CIs) for infertility as the outcome adjusting for age, BMI, SES, and physical activity. P-values for trends were calculated across DII quartiles. All statistical analyses were performed using STATA version 14.1 (Stata Corp, College Station, TX, USA). A P-value < 0.05 was considered statistically significant.

## Results

Among 4437 women enrolled in this study, 411 (9.26%) were infertile. Almost 60% of participants were urban and 299 (12.51%) of them were infertile. Among infertile women, 252 (61.3%) of them had low physical activity (3 > MET/ h per day) which was significantly different from the fertile women (P = 0.001). (Table 1).

**Table 4:** Findings from the logistic regression models that evaluated infertility according to DII quartiles

Models	DII quartiles				P-value Linear Trend
	Odds ratios, 95% confidence intervals				
	Q1 (<-2.02)	Q2 (-2.02, -1.02)	Q3 (-1.03, 0.25)	Q4 (> 0.26)	
Crude ^a^	Ref.	0.81 (0.61–1.08)	1.14 (0.88–1.48)	1.86 (1.65–2.14)	0.001
Model 1 ^b^	Ref.	0.81 (0.61–1.08)	1.17 (0.90–1.52)	1.94 (1.71–2.25)	0.001
Model 2 ^c^	Ref.	0.77 (0.58–1.04)	1.04 (0.79–1.36)	1.76 (1.57–2.02)	0.001

**Note**: The logistic regression test was used for analysis. ^a^ Crude model: Logistic regression without adjustment; ^b^ Model 1: Logistic regression with adjustment for age; ^c^ Model 2: Logistic regression with adjustment for age, BMI, marital status, smoking, education, alcohol drinking, energy intake and physical activity.

The mean ± SD age in the infertile women was 43.67 ± 7.47 years and in the non-fertile was 48.42 ± 8.16 years. Also, the mean ± SD weight in the infertile women was 72.86 ± 13.02 kg, and in non-fertile was 70.27 ± 12.77 kg. The mean energy intake in the infertile women was 2004.5 ± 275.2 kcal/day, containing 322.9 ± 95.7 g/day carbohydrates, 49.4 ± 15.2 g/day fat, and 71.4 ± 13.2 g/day protein. According to the gram of intake from each macronutrient in infertile women, the daily percentages of carbohydrates, fat, and protein were 64.4%, 22.1%, and 14.2%, respectively (Table 2).

Table 3 summarizes the characteristics of the participants according to the quartiles of DII. The minimum and, maximum DII score was from − 2.02, (the most powerful anti-inflammatory diet) to 0.26 (the most powerful pro-inflammatory diet). There was a significant difference in the age, weight, and BMI of the participants (P < 0.001) between the DII quartiles. Also, the lipid profile including Total cholesterol (P < 0.001), LDL-C (P < 0.001), and HDL-C (P < 0.001) were significant differences between the DII quartiles. The mean total calorie intake in the fourth quartile (2135.58 ± 175.58 Kcal/d) was significantly higher than the first quartile (1877.52 ± 131.39 Kcal/d) of DII (P < 0.001). Besides, the mean energy intake from protein in the fourth quartile (391.78 ± 22.09 Kcal/d) was significantly higher than the first quartile (150.52 ± 14.06 Kcal/d) of DII (P < 0.001). However, there is no significant difference in the mean energy intake from carbohydrates and fat between the DII quartiles. In addition, the highest and lowest fiber intake was related to the first and fourth quartiles, respectively, which showed a significant difference (P < 0.001). (Table 3).

The odds of infertility in the fourth quartile of the DII (pro-inflammatory diet) is significantly 86% (OR: 1.86; 95% CI: 1.65, 2.14) higher than the first quartile (anti-inflammatory diet). After adjusting for main confounding factors including age, BMI, marital status, smoking, education, alcohol drinking, energy intake, and physical activity, the odds of infertility in the fourth quartile (pro-inflammatory) was 76% (OR: 1.76; 95% CI: 1.57–2.02) higher than first quartile (anti-inflammatory) (Table 4).

## Discussion

The present study provides evidence of an association between dietary inflammation and infertility in women. The findings of this cross-sectional study showed that having a pro-inflammatory diet increases the probability of infertility in women by 86%, which remained significant after controlling for confounding factors with odds of 76%.

Some previous investigations have examined the association between major dietary patterns and infertility. In this way, a study investigated the relationship between diet and reproductive outcomes in infertile women. In this study, three major dietary patterns have been identified (healthy, western, and unhealthy diets). Consistent with our study, they concluded that greater adherence to a healthy diet may enhance oocyte quality and quantity. On the other hand, an unhealthy diet could negatively affect the chance of pregnancy [29]. Unisa et al. 2021; conducted a study on 402,807 currently married women aged 20–49 years in India to assess the degree of primary infertility and its associated factors. At the end of the study, the results showed infertility in women is related to their diet and morbidity, and a positive influence of dark green leafy vegetables, fruits, and milk/curd was observed [30].

In addition, a randomized clinical trial showed that a healthy diet rich in fruits and vegetables, similar to the Dietary Approaches to Stop Hypertension (DASH), can have a positive effect on total antioxidant capacity (TAC). In this study, 60 women were randomly assigned to the DASH diet group (rich in fruits, vegetables, whole grains, low-fat dairy, low in saturated fat and cholesterol, refined grains, and sweets) and a control group. After 12 weeks of follow-up, women in the DASH diet group had increased TAC. Additionally, another study showed that an increase in the DASH diet was associated with an increase in plasma TAC in obese subjects with PCOS. In this study, the “healthy diet” had higher vegetables, fruits, and nuts which these food groups are rich in antioxidants [31]. It has been shown that the antioxidant capacity of plasma can be increased significantly by the antioxidants found in fruits and vegetables and it seems that a high TAC level can positively affect the percentage of oocytes and the quality of second-stage oocytes [32].

Inflammation is one of the main causes of infertility in endometriosis. It prevents decasualization, lowers progesterone levels, and disrupts endometrial function. Moreover, hormonal imbalances caused by the different diseases play a significant role in infertility, as inflammation increases aromatase activity and creates an estrogen-dominant phase. Besides, oocyte maturation and fertilization are influenced by prostaglandins and cytokines from inflammatory cells. In this way, not only the reproductive system of the woman is affected, but also the sperm entering through the uterus and fallopian tubes, their movement is reduced and their attachment to the ovary is damaged. Reactive oxygen species also damage the endometrium, Oocyte, and sperm, resulting in oxidative stress that affects the molecular level [33].

Chronic inflammation is a popular explanation of the positive association between DII and infertility, which may occur through the effect of a pro-inflammatory diet on increasing systemic inflammation [13]. Some studies have also reported that chronic inflammation plays a significant role in the risk of infertility [34]. However, we recognize that there are extensive debates among researchers about the etiology of infertility, which is multifactorial, and the only mechanism will not be whispered responsible for all cases of infertility.

The present study had some limitations in terms of methodology and nature. Firstly, the DII score was calculated using only 31 dietary parameters due to a limitation in the number of parameters. A second limitation of the present study was that, because of its cross-sectional design, there was no way to examine the causal relationship between DII and the progression of infertility. However, this study had some strengths. An important strength of this study was its large sample size, which allowed it to determine the relationship between DII and infertility among Kurdish women in Iran. Other strengths of this study are the high quality of data collection, population-based study design, and adjustment for all known confounders such as age, BMI, SES, and physical activity. As well, using DII instead of inflammatory markers to assess the effect of inflammation may help directly measure the impact of diet on clinical outcomes through inflammation and reduce the overall cost of the study. Additionally, the calculation of the DII by an inexpensive and non-invasive method (FFQ) makes it possible to evaluate the inflammatory properties of food and its relationship with some diseases. In summary, these novel findings from the cohort study provide evidence that a more pro-inflammatory diet as indexed by the DII score increased the odds of infertility. Our findings showed that a diet rich in vegetable fibers and omega-3 fatty acids and low in unhealthy dietary factors such as high simple sugars and saturated fats can be considered an appropriate approach for reducing the risk of infertility. However, more investigations are necessary to validate the kinds of conclusions that can be drawn from this study. Our data suggest that we still have a long way to go, and future research should also examine whether altering the inflammatory potential of the diet can reduce chronic inflammation and the risk of infertility.

## Data Availability

The datasets used and/or analyzed during the current study are available from the corresponding author on reasonable request.
